# Factors associated with hypoalbuminemia and malnutrition risk in older inpatients: A detailed analysis of demographic and clinical characteristics

**DOI:** 10.3934/publichealth.2025056

**Published:** 2025-11-24

**Authors:** Tran Thi Phuong Lan, Pham Van Phu, Le Thi Huong, Le Xuan Hung, Nguyen Quang Dung

**Affiliations:** 1 Department of Nutrition and Food Safety, School of Preventive Medicine and Public Health, Hanoi Medical University, 1 Ton That Tung, Dong Da, Hanoi 100000, Vietnam; 2 Department of Nutrition - C11, Military Hospital 354, 120 Doc Ngu, Ba Dinh, Hanoi 100000, Vietnam; 3 Department of Research Methodology and Biostatistics, School of Preventive Medicine and Public Health, Hanoi Medical University, 1 Ton That Tung, Dong Da, Hanoi 100000, Vietnam

**Keywords:** hypoalbuminemia, malnutrition risk, older inpatients, associated factors, demographic factors, biochemical markers, Vietnam, MNA-SF, zinc deficiency, ferritin

## Abstract

Malnutrition and hypoalbuminemia are critical health challenges among hospitalized older adults, contributing to increased morbidity, prolonged hospital stays, and higher mortality risks. In this cross-sectional study, we aimed to determine the prevalence and factors associated with hypoalbuminemia and malnutrition risk in older inpatients at Military Hospital 354, Hanoi, Vietnam, from January–June 2025. A total of 264 patients aged 65 and older were recruited from geriatric, internal medicine, and rehabilitation wards. Hypoalbuminemia was defined as serum albumin levels <35 g/L, and malnutrition risk was assessed using the Mini Nutritional Assessment-Short Form (MNA-SF; score < 12). Data on demographic (age, sex, education, cohabitation) and clinical/biochemical factors (primary diagnosis, hemoglobin, lymphocyte, zinc, ferritin) were collected within 48 hours of admission. The prevalence of hypoalbuminemia was 33.0%, and 73.5% of patients were malnourished or at risk of malnutrition. Multivariable logistic regression identified older age (*AOR* 1.09, 95% *CI* 1.01–1.18) and male sex (*AOR* 2.17, 95% *CI* 1.14–4.17) as significant factors associated with malnutrition risk, while female sex was protective for both hypoalbuminemia (*AOR* 0.48, 95% *CI* 0.27–0.86) and malnutrition risk (*AOR* 0.46, 95% *CI* 0.24–0.88). Higher lymphocyte counts were associated with hypoalbuminemia (*AOR* 1.24, 95% *CI* 1.00–1.53). Spearman's correlations revealed strong positive associations between zinc and ferritin (*r* = 0.74, *p* < 0.01) and negative correlations of age with zinc (*r* = −0.43) and ferritin (*r* = −0.33). These findings highlight the high burden of nutritional deficits in Vietnam's older inpatients and underscore the need for routine MNA-SF and albumin screening to guide early interventions, particularly targeting micronutrient deficiencies in resource-constrained settings.

## 1. Introduction

Malnutrition and hypoalbuminemia represent significant health challenges among the older adult population, particularly in hospitalized settings where they can exacerbate morbidity, prolong hospital stays, and increase mortality risks [1]–[4]. Globally, malnutrition affects up to 50% of older adults in acute care facilities, with prevalence rates often higher in developing countries due to socioeconomic factors, limited access to nutritional support, and comorbidities such as chronic obstructive pulmonary disease (COPD) and hypertension [3],[5]–[7]. Hypoalbuminemia, defined as serum albumin levels below 35 g/L, is a key biochemical marker not only for nutritional deficiency but also for systemic inflammation, liver dysfunction, and overall frailty in older adults [8],[9]. It is associated with adverse outcomes, including impaired wound healing, immune suppression, and heightened infection susceptibility [1],[10].

In Vietnam, rapid population aging, projected to see the proportion of individuals aged 65 and older rise to around 20% by 2040, has intensified the burden of age-related nutritional issues [11]. Hospitalized older adults in Vietnam face compounded risks due to dietary inadequacies, polypharmacy, and underlying chronic conditions, with studies reporting malnutrition prevalence as high as 72.2% in this group [7],[12]–[14]. Studies in Southeast Asia have highlighted associations between low serum zinc and ferritin levels with malnutrition, as these micronutrients are critical in immune function and oxidative stress response [15]–[19]. Similarly, hematological parameters like hemoglobin and lymphocyte counts are frequently diminished in malnourished states, reflecting anemia of chronic disease and immune compromise [20]–[25]. However, data on the interplay of these biomarkers with demographic factors such as age, sex, and education level remain limited in Vietnamese contexts, particularly among military hospital inpatients who may have unique exposure histories.

Despite growing recognition, few researchers have comprehensively examined the factors associated with hypoalbuminemia and malnutrition risk using multivariable models in this demographic [25],[26]. This gap underscores the need for targeted research to inform screening protocols and interventions. In this study, we aim to: (1) Determine the prevalence of hypoalbuminemia and malnutrition risk among older inpatients at Military Hospital 354 in Hanoi, Vietnam; (2) identify demographic, clinical, and biochemical factors associated with these conditions; and (3) explore correlations among key associated factors to guide future nutritional strategies. This research contributes to enhancing geriatric care in resource-constrained settings by addressing these objectives.

## 2. Materials and methods

### 2.1. Study design and setting

This cross-sectional study was conducted at Military Hospital 354, a tertiary care facility in Hanoi, Vietnam. Data were collected in January–June 2025. This hospital provides specialized care for a diverse population, including older adults with chronic and acute conditions. It is an appropriate setting for investigating factors related to nutritional and biochemical outcomes in this vulnerable group.

### 2.2. Participants and data collection

A total of 264 older inpatients were included in this study. Participants were recruited from the geriatric, internal medicine, or rehabilitation wards of Military Hospital 354.

**Inclusion criteria were:** (1) Hospital admission for ≥48 hours; (2) ability to provide informed consent or availability of a legal representative; and (3) complete data on nutritional and biochemical assessments.

**Exclusion criteria were:** (1) Terminal illness or palliative care status; (2) severe cognitive impairment (Mini-Mental State Examination score < 10) precluding reliable responses; (3) incomplete biochemical or anthropometric data; and (4) active malignancies or acute infectious diseases, to minimize confounding from severe inflammatory or catabolic states.

Data were collected within 48 hours of admission by trained dietitians and research nurses, following standardized protocols at Military Hospital 354.

### 2.3. Definition of variables

#### 2.3.1. Dependent variables

- **Hypoalbuminemia**: Defined as serum albumin levels <35 g/L. Patients were categorized as having “Hypoalbuminemia” (1) or “Normal albumin” (0) based on this threshold.
- **Malnutrition risk**: Assessed using the Mini Nutritional Assessment Short Form (MNA-SF) score. MNA-SF scores range from 0 to 14, with categorization for this study defined as “At risk/malnourished” (1) if their MNA-SF score was <12 (combining the original “malnourished” (0–7) and “at risk” (8–11) categories) or “Normal nutritional status” (0) if their score was ≥12.

#### 2.3.2. Independent variables (associated factors)

• **Demographic factors:**

 ▪ **Age**: Recorded in years.

 ▪ **Sex**: Categorized as Male or Female.

 ▪ **Education level**: Categorized as “Secondary school” (reference), or “Higher education” (combining College/University and Postgraduate levels).

 ▪ **Cohabitation status**: Categorized as “Living with family” (reference) or “Living alone”.

• **Clinical and biochemical factors:**

 ▪ **Primary diagnosis**: Categorized as hypertension, COPD, or other diagnoses. Hypertension and COPD were selected as the primary diagnostic categories due to their prevalence in the cohort, while acute or short-term illnesses were excluded for clinical consistency.

 ▪ **Length of hospital stay (LOS)**: Calculated in days from the date of hospital admission to the date of discharge.

 ▪ **Hemoglobin**: Measured in g/L.

 ▪ **hs-CRP**: Measured in serum (mg/L) using an immunoturbidimetric assay to detect low-grade systemic inflammation.

 ▪ **Lymphocyte**: Measured as an absolute count (×10^9^/L).

 ▪ **Zinc**: Measured in µmol/L.

 ▪ **Ferritin**: Measured in µg/L.

### 2.4. Ethical considerations

The study protocol was approved by the Ethics Committee of Hanoi Medical University (Permit: 1294/GCN–HMUIRR, dated December 15, 2024). Written informed consent was obtained from all participants or their legal representatives before enrollment. Data were handled with strict confidentiality, and potentially identifying information was secured.

### 2.5. Statistical analysis

Data were analyzed using R statistical software (R Foundation for Statistical Computing, Vienna, Austria). Descriptive statistics were used to summarize continuous variables as means ± standard deviations (*SD*) and medians with interquartile ranges (*IQR*), while categorical variables were presented as frequencies (*n*) and percentages (%). Univariate analyses were conducted to compare demographic and clinical characteristics between groups (e.g., Hypoalbuminemia *vs*. Normal albumin, at risk/malnourished *vs*. Normal nutrition); Chi-square tests were applied for categorical variables; and independent *t*-tests or Mann-Whitney *U* tests were used for continuous variables, depending on data distribution. To assess potential multicollinearity, Spearman's rank correlation was employed to examine relationships among continuous clinical and biochemical variables. Two separate multivariable logistic regression models were fitted to identify independent associated factors for Hypoalbuminemia (binary outcome: Yes/No) and Malnutrition risk (binary outcome: At risk/malnourished Yes/No), with associated factors including Age, Sex, Education level, Hemoglobin, Lymphocyte, Zinc, and Ferritin. Adjusted Odds Ratios (*AORs*), 95% Confidence Intervals (*CIs*), and p-values were reported. CRP and LOS were excluded from multivariable models to avoid collinearity with acute-phase responses and disease severity, while primary diagnosis categories had limited sample sizes for stable model estimation. Observations with missing data were excluded from specific analyses (complete case analysis). A two-tailed *p*-value < 0.05 was considered statistically significant.

## 3. Results

We enrolled 264 older inpatients at Military Hospital 354, Hanoi, Vietnam. Detailed demographic and clinical characteristics of the study participants, stratified by hypoalbuminemia and malnutrition risk status, are presented in Table 1. Participants with hypoalbuminemia were significantly older (74.25 ± 4.01 years) compared to those with normal albumin (72.97 ± 4.13 years; *p* = 0.013). Similarly, patients at risk/malnourished were substantially older (73.78 ± 4.04 years) than those with normal nutritional status (71.96 ± 3.99 years; *p* = 0.002). Notably, individuals with hypoalbuminemia exhibited significantly lower mean Hemoglobin (123.84 ± 20.66 *vs*. 128.65 ± 21.49 g/L; *p* = 0.045) and Zinc (88.40 ± 22.12 *vs*. 97.01 ± 21.96; *p* = 0.006) levels compared to those with normal albumin. For malnutrition risk, a statistically significant difference was observed in Primary diagnosis (*p* = 0.023), with 23.9% of normally nourished patients having Hypertension compared to 10.4% in the at-risk/malnourished group. Furthermore, at-risk/malnourished individuals had significantly lower mean Lymphocyte (2.04 ± 1.68 *vs*. 2.14 ± 0.95; *p* = 0.044) and Ferritin (115.90 ± 55.84 *vs*. 130.99 ± 59.16; *p* = 0.036) levels compared to normally nourished patients.

Multivariable logistic regression models were fitted to identify independent factors associated with hypoalbuminemia and malnutrition risk, with results in Table 2. In the hypoalbuminemia model, significant associated factors included sex and Lymphocyte levels. Females had significantly lower odds of hypoalbuminemia compared to males [Adjusted Odds Ratio (*AOR*) = 0.48, 95% *CI* (0.27 to 0.86), *p* = 0.013]. Conversely, higher Lymphocyte levels were associated with significantly increased odds of hypoalbuminemia [*AOR* = 1.24, 95% *CI* (1.00 to 1.53), *p* = 0.046].

In the malnutrition risk model, age and sex were identified as significant associated factors. Older age was associated with significantly increased odds of being at risk/malnourished [*AOR* = 1.09, 95% *CI* (1.01 to 1.18), *p* = 0.036]. Similar to hypoalbuminemia, females had significantly lower odds of malnutrition risk than males [*AOR* = 0.46, 95% *CI* (0.24 to 0.88), *p* = 0.019].

The prevalence of hypoalbuminemia and malnutrition risk across key demographic and clinical subgroups is presented in Table 3. Overall, 33.0% of the study participants exhibited hypoalbuminemia, and 73.5% were categorized as at risk or malnourished.

**Table 1.** Baseline characteristics of study participants by hypoalbuminemia and malnutrition risk status.

**Characteristic**	**Hypoalbuminemia** (<35 g/L) (*N* = 87)	**Normal** (≥35 g/L) (*N* = 177)	**At risk/malnourished** (<12) (*N* = 192)	**Normal** (12–14) (*N* = 67)
**Age (years),** mean ± *SD*	74.25 ± 4.01	72.97 ± 4.13	73.78 ± 4.04	71.96 ± 3.99
**Length of Hospital Stay (days),** median (*IQR*)	6 (2–12)	6 (4–7)	4 (1–10)	-
**Sex, *n* (%)**				
Male	50 (57.5%)	83 (46.9%)	101 (52.6%)	27 (40.3%)
Female	37 (42.5%)	94 (53.1%)	91 (47.4%)	40 (59.7%)
**Primary diagnosis,** *n* (%)				
Hypertension	11 (12.6%)	25 (14.1%)	20 (10.4%)	16 (23.9%)
COPD	12 (13.8%)	11 (6.2%)	17 (8.9%)	5 (7.5%)
Other	64 (73.6%)	141 (79.7%)	155 (80.7%)	46 (68.7%)
**Education level,** *n* (%)				
Secondary school	35 (40.2%)	66 (37.3%)	77 (40.1%)	21 (31.3%)
College/University	42 (48.3%)	95 (53.7%)	95 (49.5%)	40 (59.7%)
Postgraduate	10 (11.5%)	16 (9.0%)	20 (10.4%)	6 (9.0%)
**Cohabitation status,** *n* (%)				
Living with family	86 (98.9%)	175 (99.4%)	189 (99.0%)	67 (100.0%)
Living alone	1 (1.1%)	1 (0.6%)	2 (1.0%)	0 (0.0%)
**Hemoglobin,** mean ± *SD*	123.84 ± 20.66	128.65 ± 21.49	127.20 ± 19.14	126.83 ± 27.29
**hs-CRP,** median (*IQR*)	14 (2–34)	22 (3–61)	27 (2–60)	9 (6–25)
**Lymphocyte,** mean ± *SD*	2.28 ± 2.31	1.94 ± 0.88	2.04 ± 1.68	2.14 ± 0.95
**Zinc,** mean ± *SD*	88.40 ± 22.12	97.01 ± 21.96	92.43 ± 22.30	100.42 ± 21.53
**Ferritin,** mean ± *SD*	109.91 ± 50.41	124.84 ± 59.07	115.90 ± 55.84	130.99 ± 59.16

Note: All biochemical markers were measured in serum. Units: albumin (g/L), hemoglobin (g/L), lymphocyte (×10⁹/L), zinc (µmol/L), ferritin (µg/L), hs-CRP (mg/L). “-” indicates data not available for this subgroup.

**Table 2.** Multivariable logistic regression models for hypoalbuminemia and malnutrition risk in older inpatients.

**Associated factors**	**Hypoalbuminemia model** [*AOR* (95% *CI*), *p*]	**Malnutrition risk model** [*AOR* (95% *CI*), *p*]
**Age (years)**	1.07 (0.99 to 1.15), *p* = 0.104	1.09 (1.01 to 1.18), ***p* = 0.036**
**Hemoglobin**	0.99 (0.98 to 1.00), *p* = 0.178	1.00 (0.99 to 1.02), *p* = 0.529
**Lymphocyte**	1.24 (1.00 to 1.53), ***p* = 0.046**	0.95 (0.81 to 1.10), *p* = 0.466
**Zinc**	0.99 (0.97 to 1.01), *p* = 0.181	0.99 (0.97 to 1.01), *p* = 0.369
**Ferritin**	1.00 (0.99 to 1.01), *p* = 0.829	1.00 (0.99 to 1.00), *p* = 0.396
Sex: Female *vs*. Male	0.48 (0.27 to 0.86), ***p* = 0.013**	0.46 (0.24 to 0.88), ***p* = 0.019**
**Education level:**
Higher education *vs*. Secondary school	1.10 (0.62 to 1.97), *p* = 0.738	0.77 (0.41 to 1.43), *p* = 0.403

**Table 3.** Prevalence of hypoalbuminemia and malnutrition risk across key demographic and clinical subgroups.

**Characteristic**	**Hypoalbuminemia,** *n* (%)	***P*-value** (Albumin)	**Malnutrition risk,** *n* (%)	***P*-value** (Nutrition)
**Age Group (years)**		**0.008**		**0.015**
65–69	12 (24.0%)		29 (58.0%)	
70–74	26 (25.7%)		74 (74.0%)	
75–79	43 (46.2%)		74 (80.4%)	
80+	6 (30.0%)		15 (88.2%)	
**Sex**		0.138		0.111
Male	50 (37.6%)		101 (78.9%)	
Female	37 (28.2%)		91 (69.5%)	
**Primary diagnosis**		0.121		**0.023**
Hypertension	11 (30.6%)		20 (55.6%)	
COPD	12 (52.2%)		17 (77.3%)	
Other	64 (31.2%)		155 (77.1%)	
**Education level**		0.743		0.260
Secondary school	35 (34.7%)		77 (78.6%)	
College/University	42 (48.3%)		95 (49.5%)	
Postgraduate	10 (11.5%)		20 (10.4%)	
**Cohabitation status**		0.686		0.589
Living with family	86 (33.0%)		189 (73.8%)	
Living alone	1 (50.0%)		2 (100.0%)	
Unknown/Not specified	0 (0.0%)		1 (100.0%)	

Significant variations in prevalence were observed across several subgroups. Hypoalbuminemia prevalence varied significantly by age group (*p* = 0.008), notably increasing from 24.0% in the 65–69 age group to 46.2% in the 75–79 age group. Malnutrition risk also demonstrated a statistically significant increase with age (*p* = 0.015), rising from 58.0% in the 65–69 age group to 88.2% in the 80+ age group. Regarding primary diagnoses, the prevalence of malnutrition risk showed a statistically significant difference (*p* = 0.023), with patients diagnosed with COPD (77.3%) and other diagnoses (77.1%) exhibiting higher rates compared to those with hypertension (55.6%). No statistically significant differences in prevalence were observed across sex, education level, or cohabitation status in this univariate analysis.

Spearman's rank correlations were performed to examine the relationships among continuous clinical and biochemical variables, with results presented in Table 4. Age showed significant negative correlations with Zinc (*r* = −0.43, *p* < 0.01) and Ferritin (*r* = −0.33, *p* < 0.01), and a weaker negative correlation with Lymphocyte (*r* = −0.14, *p* < 0.05). Hemoglobin exhibited significant positive correlations with Lymphocyte (*r* = 0.21, *p* < 0.01), zinc (*r* = 0.38, *p* < 0.01), and ferritin (*r* = 0.48, *p* < 0.01). Zinc and ferritin were positively correlated (*r* = 0.74, *p* < 0.01).

**Table 4.** Spearman's rank correlations among continuous clinical and biochemical variables.

**Variable**	**Age**	**Hemoglobin**	**Lymphocyte**	**Zinc**	**Ferritin**
**Age**	-	−0.10	−0.14*	−0.43**	−0.33**
**Hemoglobin**	−0.10	-	0.21**	0.38**	0.48**
**Lymphocyte**	−0.14*	0.21**	-	0.16**	0.16*
**Zinc**	−0.43**	0.38**	0.16**	-	0.74**
**Ferritin**	−0.33**	0.48**	0.16*	0.74**	-

Note: * *p* < 0.05, ** *p* < 0.01.

## 4. Discussion

We investigated the prevalence, associated factors, and interrelationships of hypoalbuminemia and malnutrition risk among older inpatients at a tertiary military hospital in Hanoi, Vietnam. Our findings revealed a substantial burden of these conditions, with 33.0% of participants exhibiting hypoalbuminemia (serum albumin <35 g/L) and 73.5% at risk of or experiencing malnutrition (MNA-SF score < 12). This high prevalence likely reflects the combined effects of advanced age, chronic comorbidities, such as COPD and hypertension, and limited pre-admission nutritional support. These results align with our primary aim of determining prevalence in this vulnerable population and underscore the urgent need for routine nutritional screening in geriatric care settings [4],[5],[19].

The observed prevalence of malnutrition risk (73.5%) is notably high, consistent with reports from other Vietnamese hospital settings, where rates of malnutrition or risk thereof among older inpatients range from 71.6% to 72.2% [7],[12],[13]. Globally, malnutrition affects 20%–50% of hospitalized older adults, with higher figures in resource-limited regions or among those with comorbidities like COPD or hypertension, as seen in our cohort [3],[4],[25],[26]. The elevated rate in our study may reflect the unique profile of military hospital patients, who have chronic conditions exacerbated by historical exposures, alongside Vietnam's rapid population aging, where the proportion of individuals aged 65+ is projected to reach nearly 20% by 2040 [11],[14]. Hypoalbuminemia prevalence (33.0%) was lower than in some studies reporting up to 87% in hospitalized older adults, potentially due to our stricter threshold (<35 g/L) or differences in patient acuity [1],[2],[8],[9]. Nonetheless, this figure highlights hypoalbuminemia as a standard marker of nutritional compromise and inflammation in this group [9],[10].

Addressing the second aim, we identified key demographic, clinical, and biochemical factors associated with hypoalbuminemia and malnutrition risk. Older age was significantly associated with malnutrition risk (*AOR* 1.09, 95% *CI* 1.01–1.18), with prevalence rising from 58.0% in those aged 65–69 to 88.2% in those 80+, mirroring trends in other studies where advancing age correlates with heightened vulnerability due to reduced intake, polypharmacy, and comorbidities [9],[13],[18],[26]. Interestingly, age trended toward significance for hypoalbuminemia (*AOR* 1.07, *p* = 0.104), suggesting it may act indirectly through inflammatory pathways or nutritional decline [1],[10]. Female sex was protective against both conditions (*AOR* 0.48 for hypoalbuminemia; *AOR* 0.46 for malnutrition risk), contrasting some reports of higher malnutrition in females but aligning with others indicating males' greater susceptibility, possibly due to lifestyle factors or muscle mass differences in Asian populations [7],[18],[23].

Clinical diagnoses influenced malnutrition risk, with higher rates in COPD (77.3%) and other diagnoses (77.1%) versus hypertension (55.6%), supporting evidence that respiratory and multisystem diseases exacerbate nutritional deficits through increased catabolism and appetite suppression [13],[25],[26]. Biochemical factors were prominent: Participants with hypoalbuminemia had lower hemoglobin and zinc, while those at malnutrition risk showed reduced lymphocyte and ferritin levels. These align with literature linking anemia (low hemoglobin) to malnutrition in older adults, often as an indicator of iron deficiency or chronic inflammation [20]–[22],[24]. In Southeast Asia, zinc deficiency impairs immune function and appetite, contributing to malnutrition cycles [15]–[17]. Lower ferritin may reflect iron stores depletion amid inflammation, while reduced lymphocytes signal immune compromise, a hallmark of malnutrition [6],[20],[24]. Inflammation likely played a mediating role in these associations. The inclusion of hs-CRP in the dataset provided additional context for identifying low-grade inflammatory activity, although this variable was excluded from the final regression model due to collinearity. Elevated hs-CRP levels observed in our cohort support the view that inflammatory responses may underlie hypoalbuminemia and nutritional deterioration in older adults. Another notable finding was the borderline association between lymphocyte count and hypoalbuminemia (*AOR* = 1.24, 95% *CI* 1.00–1.53; *p* = 0.046). Although this association met the threshold for statistical significance, the confidence interval narrowly included unity and therefore should be interpreted with caution. This borderline finding likely reflects acute inflammatory or immune-reactive processes rather than a causal nutritional effect. Inflammatory responses can transiently elevate lymphocyte levels while reducing serum albumin as part of the negative acute-phase reaction. This suggests that both parameters are influenced by shared inflammatory pathways rather than a direct nutritional mechanism. Future longitudinal or mechanistic studies are warranted to determine whether this relationship represents compensatory immune activation or residual confounding from infection or disease severity [20],[24].

Fulfilling the third aim, Spearman's correlations revealed strong positive associations between zinc and ferritin (*r* = 0.74, *p* < 0.01), suggesting shared pathways in micronutrient metabolism and inflammatory response [15],[16]. Negative correlations of age with zinc (*r* = −0.43) and ferritin (*r* = −0.33) highlight age-related declines in these nutrients, potentially exacerbating frailty [16],[22]. Positive links between hemoglobin and ferritin (*r* = 0.48) underscore iron's role in anemia prevention [6],[15],[21]. These interrelationships guide targeted interventions, such as micronutrient supplementation, to disrupt vicious cycles of deficiency and poor outcomes [15]–[17].

These findings have implications for geriatric care in Vietnam, where malnutrition contributes to prolonged stays, infections, and mortality [4],[14],[18],[25]. Integrating MNA-SF and albumin screening into admission protocols could enable early interventions like oral supplements or multidisciplinary support, potentially improving quality of life [4],[12],[13]. Addressing micronutrient gaps, particularly zinc and iron, aligns with regional recommendations for Southeast Asia [15]–[17].

Limitations include the cross-sectional design, which limits causal inference, and the potential overestimation of nutritional risk by MNA-SF in acute illness [4]. As a single-center study in a military hospital, the findings may not generalize to civilian settings. Excluding variables such as hs-CRP, length of stay, and primary diagnosis to avoid collinearity may have led to residual confounding. Furthermore, complete-case analysis for missing data could introduce bias. Additionally, blood pressure and oxygen saturation were not systematically recorded, limiting the evaluation of their associations with nutritional outcomes among hypertensive and COPD patients. Despite these constraints, the findings provide valuable baseline evidence. Future multi-center prospective studies incorporating comprehensive nutritional and inflammatory assessments and integration of MNA-SF with GLIM criteria are warranted to validate these associations and explore outcomes such as post-discharge frailty [13],[26].

## 5. Conclusions

This study demonstrates a high prevalence of hypoalbuminemia (33.0%) and malnutrition risk (73.5%) among older inpatients at Military Hospital 354 in Hanoi, Vietnam, aligning with regional and global patterns in hospitalized older adults. Key associated factors include older age and male sex associated with higher malnutrition risk, whereas female sex appeared protective against both conditions; biochemical markers such as lower hemoglobin, zinc, and ferritin levels, and altered lymphocyte counts further underscore the multifactorial nature of these nutritional deficits. Correlations among associated factors, notably strong positive associations between zinc and ferritin, highlight interconnected micronutrient pathways that exacerbate age-related vulnerabilities.

By addressing the study aims, our findings contribute to understanding nutritional challenges in Vietnam's aging population, which is projected to grow significantly by 2040. Routine screening with tools like MNA-SF and serum albumin and targeted interventions for micronutrient deficiencies (e.g., zinc and iron supplementation) could mitigate adverse outcomes, including prolonged hospital stays, frailty, and mortality. Future research should prioritize prospective designs and broader cohorts to evaluate intervention efficacy in resource-constrained settings.

## Use of AI tools declaration

The authors used assistive AI language tools for grammar and clarity only; no generative content was produced. The authors take full responsibility for the content.
